# Pollination ecotypes and the origin of plant species

**DOI:** 10.1098/rspb.2024.2787

**Published:** 2025-01-29

**Authors:** Steven D. Johnson

**Affiliations:** ^1^ Centre for Functional Biodiversity, University of KwaZulu-Natal, Pietermaritzburg 3209, South Africa

**Keywords:** local adaptation, floral syndromes, mutualism, range limits, pollination, speciation

## Abstract

Ecological niche shifts are a key driver of phenotypic divergence and contribute to isolating barriers among lineages. For many groups of organisms, the history of these shifts and associated trait–environment correlations are well-documented at the macroevolutionary level. However, the processes that generate these patterns are initiated below the species level, often by the formation of ecotypes in contrasting environments. Here, I review the evidence in plants for ‘pollination ecotypes’ as microevolutionary responses to environmental gradients in pollinator availability. Pollinators are critical for population establishment and persistence in most species, thereby forming part of their fundamental niche. Novel floral trait combinations allow species to exploit particular pollination opportunities in local habitats and evolve primarily through sexual selection due to their effects on mating success. I examine selected case studies on the evolution of pollination ecotypes, including self-pollinating forms, and use these to illustrate challenging practical and conceptual issues. These issues include the paucity of reliable natural history data, the problem of implementing and interpreting reciprocal translocation experiments, and establishing criteria for when allopatric ecotypes should be considered species.

## 1. Introduction

The ecotype concept was first proposed just over 100 years ago by Göte Turesson [1] to explain intraspecific trait divergence associated with shifts among ecological niches. Turesson used common garden experiments to establish the genetic basis of trait divergence in a range of plant species occurring along environmental gradients in Sweden. Later, Jens Clausen and co-workers [2,3] pioneered the use of reciprocal translocation (transplant) experiments to confirm the role of local adaptation in the divergence of traits across environmental gradients in California. Numerous subsequent studies have demonstrated both climatic and edaphic ecotypes in plants [4–6], and the ecotype concept is increasingly adopted in animal studies [7]. The concept has enjoyed a resurgence due to the widespread acceptance of ecological speciation as the underlying basis for adaptive radiations in plants and animals [8–10]. The formation of ecotypes is now widely considered an intermediate stage in speciation [5,11,12]. Because divergence among ecotypes is, on average, more recent than that among sister species, the current environments that ecotypes occupy are more likely to represent conditions at the time of divergence [13]. Studying ecotypes with incomplete reproductive isolation can also facilitate the detection of genetic signatures of divergent selection [14]. Thus, studies of ecotypes provide particularly valuable insights into the environmental drivers of phenotypic divergence, and ultimately, of speciation [15]. Following Charles Darwin, Verne Grant emphasized the value of studies on intraspecific variation, as it allows speciation to be studied ‘as a process, rather than as a historical event’ [16, p. 162]. But how and why some ecotypes transition to the species level remain major questions in evolutionary biology [5,17].

Geographical trait variation below the species level is very common in plants and animals. This variation is usually recognized either in formal taxonomy, as subspecies or varieties, or, *inter alia*, as a species complex, geographical race, semi-species, incipient species, morphotype, ecological race or ecotype. Discerning whether geographical variation results from genetic differences, plasticity or both, and whether it reflects local adaptation, neutral processes or pleiotropy formed the basis for the research agendas of Turesson, Clausen and others [18], and remains key problems in research on population divergence.

In plants, the ecotype concept has been applied mostly to population diversification in vegetative traits, such as growth form and leaf morphology, that are expected to promote survival and growth in different environments [4]. The application of the concept to cases of floral divergence has been more recent and much more tentative. Herbert Baker [19], in an address to a meeting of the Royal Society of New Zealand in 1979, seems to have first referred to ‘pollination ecotypes’. Nevertheless, Grant and other earlier workers during the biosystematics era recognized the importance of local adaptation of plants to pollinators in intraspecific divergence of floral traits; however, they tended to refer to such cases as ecological or geographical ‘races’ [16,20]. Authors such as Grant and Ledyard Stebbins likely wanted to avoid controversies that surrounded the ecotype concept, especially concerning the application of the concept to species with clinal variation [4,5]. Furthermore, many evolutionary biologists in the twentieth century, including Theodosius Dobzhansky [21] and Clausen [22], considered ecotypes and ecological races to be essentially synonymous and both terms continue to be used fairly interchangeably. From the 1990s onwards, the term ‘pollination ecotype’ started to appear in research papers [23]. In addition, Armbruster [24] introduced the similar term ‘reproductive ecotypes’ and emphasized the importance of the legacy of Turesson and Clausen.

In this Darwin review, I examine the role of local adaptation to pollinators during the evolution of ‘pollination ecotypes’. The literature on this topic is relatively small (compared with edaphic and climate ecotypes), but it is growing rapidly and has important implications for understanding how pollinators contribute to angiosperm radiation, particularly the diversification of floral traits and the evolution of reproductive isolation among lineages [25,26]. I begin by building a case for pollinators and community-level interactions among plants and pollinators as components of fundamental and realized ecological niches for plant reproduction, respectively, and for a niche-based perspective on floral trait syndromes. I then examine evidence for geographical mosaics of pollinator availability and review some case studies of divergence in floral traits in response to these gradients. Finally, I discuss the implications of microevolutionary studies of floral trait divergence for understanding the nature of species and the process of speciation, focusing particularly on whether ecotypes represent key intermediate stages in ecological speciation.

## 2. The pollination niche and floral trait syndromes

Niches are ecological opportunities for organisms and include a ‘fundamental’ component necessary for population establishment and a ‘realized’ component arising from interactions among organisms such as competition and facilitation [27]. Traits that enable organisms to exploit these opportunities are favoured by selection, resulting in correlations between the traits and environment [8]. Trait–environment correlations are also evident in patterns of convergence among unrelated organisms that occupy similar niches [28]. Shifts among different ecological niches are now widely accepted as the basis of most evolutionary radiations [8,29]. A key reason why organisms become specialized for occupation of particular ecological niches is the trade-offs imposed by trait deployment [30,31]. A trait that increases the fitness of an organism in a particular niche will often decrease fitness in another [30,31]. Consequently, even ecological generalists exhibit some evolutionary specialization [31].

Pollen vectors represent a key dimension of the ecological niches of plants as they provide opportunities for reproduction [32,33]. Floral traits that enable plants to exploit locally effective vectors are favoured by sexual selection acting through increased mating success [34]. Such selection may generate syndromes of floral traits that are characteristically associated with different pollen vectors and, for animal-pollinated species, mechanisms for exploiting different body parts of pollinators for pollen transfer [20,35]. Even floral ‘selfing syndromes’ [36] that enable plants to reproduce in the absence of pollinators may be shaped by the local availability of pollen vectors [37]. Floral ‘pollination syndromes’ are particularly clear examples of trait–environment correlation and testify to the importance of the pollination niche dimension for the evolution of floral traits and to the general importance of ecological speciation driven by pollinators [32]. This dimension is hereafter identified as the ‘pollination niche’ and pollen vectors as ‘pollinators’.

That pollinators constitute part of the fundamental niche of plants was first emphasized by Baker & Hurd [38, p. 399] who drew attention to ‘restrictions which are placed upon the geographical distributions of plants by the lack of availability of suitable pollinators’. They cited several examples, such as the coincident distribution of macroglossine bats and bat-pollinated plants on Pacific islands. Plants with highly specialized pollination systems rarely become invasive in new geographical regions unless they can self-fertilize facultatively [39]. In addition, niche modelling that accounts for pollinator distributions can characterize the distributions of plants with specialized pollinators more accurately than is the case when only abiotic variables are used [40].

Realized pollination niches are shaped at the community level through interactions among organisms such as competition and facilitation [24,41–43]. From this perspective, the niche for plant mating is usually the particular set of local pollinators together with the guild of plants with which they interact [32]. Plant adaptation to the fundamental pollination niche can result in several outcomes, including matching of floral traits to the morphological, sensory and cognitive characteristics of local pollinators, while adaptation to the realized pollination niche can include floral food-source mimicry and floral modifications that result in pollen being placed on different pollinator body parts [24,41,43–45]. Realized pollination niches can sometimes be identified from modularity (particularly frequent interactions of groups of species) within community-level interaction networks [33,46].

The sexual displays of animals and plants evolve mostly through sexual selection for traits that increase mating success [47,48]. In animals, sexual selection often proceeds via innate female preferences for exaggerated male traits and competition among males based on display traits [48]. In some animals, the environment can modulate this selection, such as when habitat influences the acoustics of mating calls or susceptibility to predation [49]. For plants, the environment pervasively affects sexual selection when pollinators mediate the influence of floral traits on mating success. Consequently, differences among pollination environments promote the divergence of floral traits of plants through sexual selection. Interestingly, the indirect mating of plants in which pollen vectors are involved precludes Fisherian runaway sexual selection and should stabilize sexual selection compared to that in most animals. In most cases, plants adapt to the pre-existing sensory biases and morphology of pollinators [50]. Nevertheless, escalatory coevolution between plants and pollinators can lead to novel pollinator niches and this can drive floral trait diversification [51–53].

## 3. Pollinator shifts below the species level

Ecotype formation is usually triggered by the expansion of a species’ range into new environments. For example, in a seminal study, Grant & Grant [16] showed that *Saltugilia* (formerly *Gilia*) *splendens* (Polemoniaceae) had diverged into four pollination ecotypes (which they termed geographical races) in the western USA. These ecotypes vary according to flower size, shape and herkogamy (stigma–anther separation), depending on whether they are pollinated mainly by various flies, hummingbirds or self-pollinate autonomously. The Grants argued that geographical variation in the ‘pollinator climate’ drove this evolutionary diversification. Echoing their views, Stebbins [20, p. 320] later wrote that ‘The evolutionary shift from one vector to another is probably triggered by the entrance of the plant into a habitat where the original vector is scarce and the new vector is abundant’.

Extreme niche specialization can inhibit shifts among niches because of trade-offs. Stebbins [20] recognized that this constraint also applied to pollinator shifts. He speculated that such shifts involved ‘selection along lines of least resistance’, meaning that a shift to new pollinators that utilize existing floral traits would be more likely than to pollinators that required an entirely new set of traits. Consequently, Stebbins proposed that pollinator shifts should typically entail an ‘intermediate stage of double function’ involving some overlap in pollinators during intermediate stages. This hypothesis is consistent with evidence for ‘bimodal pollination’ by two different functional pollinator groups [33], or even by both animal and wind pollination (ambophily) in some plant species [54]. For plants with highly specialized pollination systems, such as sexual deception of male Hymenoptera or brood-site mutualisms, shifts tend to involve pollinator species belonging to the same functional groups, probably owing to the extreme specialization of floral traits [45]. In general, shifts among functionally different pollinators should be most frequent in plant lineages with intermediate floral specialization, facultative (delayed) selfing mechanisms, or both. Such plants can reproduce outside the range of particular pollinators and yet also respond to divergent selection when encountering new vectors.

Studies of edaphic and climatic ecotypes have shown evidence of repeated parallel evolution of ecotypes (e.g. [55]). In contrast, the evidence for this phenomenon in pollination ecotypes remains limited and mostly confined to selfing forms in otherwise outcrossing species [56–58], although a case of parallel evolution of long-tubed floral ecotypes is evident in a species of South African Iridaceae [59]. The direction of pollination niche shifts has also been established by several ecotype studies that have incorporated phylogenetic data [56,60].

## 4. Environmental gradients in pollinator availability

Unlike edaphic mosaics or geographical and seasonal gradients of temperature and rainfall, the ‘pollinator climate’ *sensu* Grant [16] is not evident to the casual observer and is remarkably difficult to document. Spatial and temporal gradients of pollinator availability result from a complex of factors, ranging from pollinator physiology to their interactions with other organisms.

Few studies have thoroughly documented the underlying ecological gradients in pollinator assemblages that gave rise to divergence in floral traits. Surveys of flower visitors can introduce an element of circularity owing to ecological sorting of flower visitors by floral traits [61] but can be informative about the distribution of realized niches among communities [33,46]. Ideally, the pollinator dimension of fundamental niches should be identified from known distributions of pollinators based on museum records or contemporary ecological surveys [62,63].

### (a) Geographical gradients

The geographical distribution of pollinator niches may be due to the effects of temperature and precipitation on pollinator physiology or other ecological factors, such as the dependence of pollinators on particular food sources. Geographical gradients in abundance and species composition are evident for many pollinator groups, including bats [64], birds [60,63], bees [65], long-proboscid flies [26,51,53] and hawkmoths [62].

A widespread geographical pattern of pollinator availability involves the depauperate pollinator fauna on islands [66,67]. For example, the absence of large native bees, the general rarity of long-tongued pollinators in New Zealand (Webb and Kelly 1993) and the complete absence of native bees on some oceanic islands appear to have shaped local floral evolution [68].

### (b) Elevational gradients

Ecological gradients from bee-dominated pollinator assemblages at lower elevations to fly-dominated assemblages at higher elevations have been reported from several continents and appear to be governed by temperature [69–72]. These gradients have important implications for floral evolution. For example, species at higher elevations commonly have bowl- or disc-shaped flowers, whereas lower elevation species are more likely to have zygomorphic flowers that restrict nectar access to long-tongued pollinators [73].

Elevational gradients also influence hummingbird and hawkmoth abundance. In Mexico, Cruden [74] found that hummingbird-pollinated plants performed better than bee-pollinated plants above 2300 m during the rainy season when cloud cover is common, whereas the converse occurred at mid-elevation sites. He also showed that the frequency of moth-pollination (based on moth scales and pollen on stigmas) decreased with elevation in North America, which he attributed to temperature effects on flight activity [75].

### (c) Fine-scale gradients

**Figure 1.**
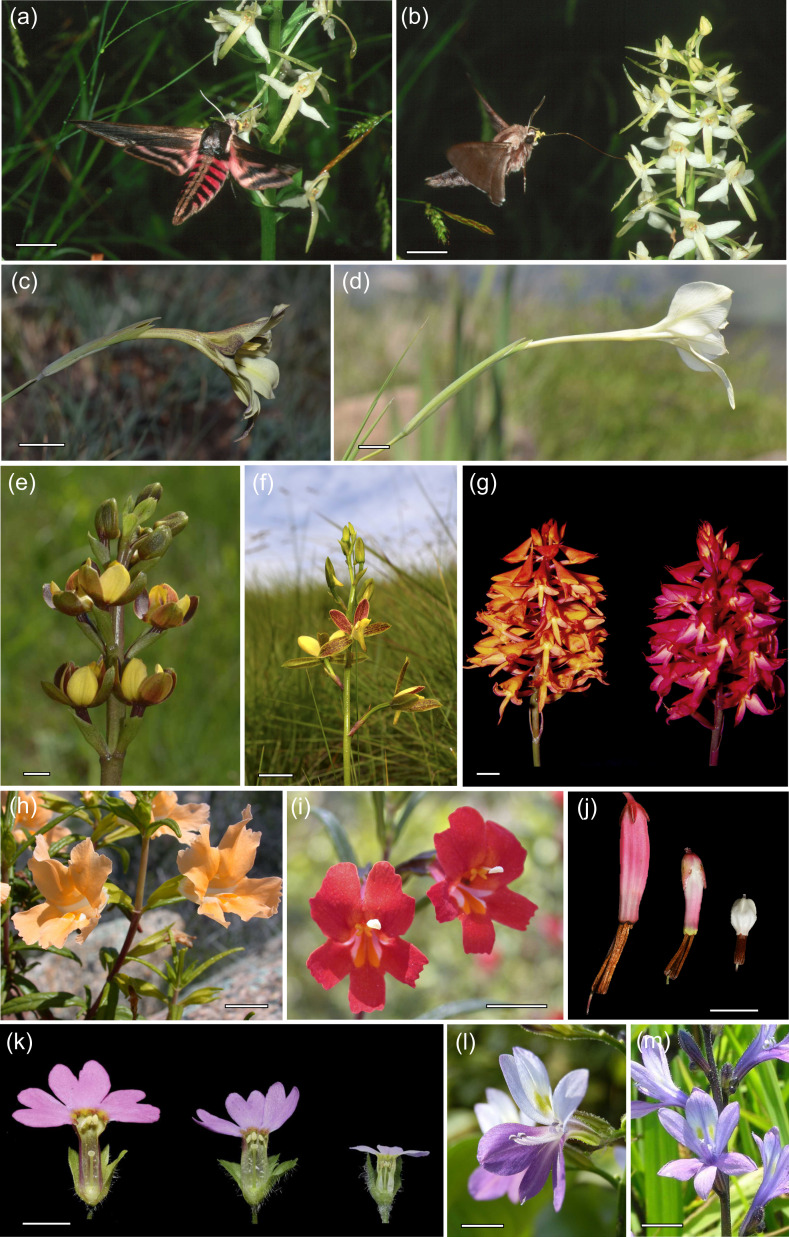
Examples of pollination ecotypes. (*a,b*) *Platanthera bifolia* (Orchidaceae); long-spurred ecotype (*a*) pollinated by the hawkmoth *Sphinx ligustri* and shorter-spurred ecotype (*b*) pollinated by the hawkmoth *Hyloicus pinastri* [76]. (*c,d*) *Gladiolus longicollis* (Iridaceae); short-tubed form (*c*) pollinated by short-proboscid hawkmoths and long-tubed ecotype (*d*) pollinated by long-proboscid hawkmoths [62]. (*e,f*) *Eulophia parviflora* (Orchidaceae); spurless ecotype pollinated by beetles (*e*) and spurred ecotype (*f*) pollinated by solitary bees [77]. (*g,h*) *Disa ferruginea* (Orchidaceae); butterfly-pollinated orange-flowered ecotype (left) and red-flowered ecotype (right) [78]. (*h,i*) *Mimulus aurantiacus*. (Phrymaceae); yellow-flowered ecotype pollinated by hawkmoths (*h*) and red-flowered ecotype pollinated by hummingbirds [63]. (*j*) *Erica plukenetii* (Ericaceae); long-tubed ecotype pollinated by malachite sunbirds (left), medium-tubed ecotype pollinated by orange-breasted sunbirds (centre) and short-spurred ecotype pollinated by noctuid moths (right) [60]. (*k*). *Primula oreodoxa* complex (Primulaceae); *P. oreodoxa* short-styled morph (left), *P. oreodoxa* homostylous morph (centre) and homostylous *P. dumicola* (right) [37]. (*l,m*) *Eichhornia paniculata* (Pontederiaceae); short-styled morph (*l*) and semi-homostylous morph (*m*) [79]. Scale bars (*a–j*) = 10 mm, (*k–m*) = 5 mm. Photo credits: Steve Johnson (*a–g*), Matt Streisfeld (*h,i*), Timo van der Niet (*j*), Wei Zhou (*k*) and Spencer Barrett (*l,m*).

Pollinator assemblages can change markedly from one vegetation type to another and have implications for the evolution of parapatric ecotypes [61]. The maintenance of pollination ecotypes of the orchid *Platanthera bifolia* in Scandinavia (figure 1a,b), for example, has been attributed to divergent selection due to differences in hawkmoth assemblages between broad-leaved deciduous forest, coniferous forest and open meadows [76].

### (d) Temporal gradients

Seasonal or diel activity patterns of pollinators can create geographically structured temporal gradients of pollinator availability, such as the spring emergence of queen bumblebees at northern latitudes, which creates a niche for generalized food deception, and early emergence of male solitary bees and wasps in Mediterranean-climate regions, which is associated with sexual deception in spring-flowering orchids [45]. In temperate North America, seasonal migration of hummingbirds creates temporal niches for the evolution of bird-pollinated flowers [16]. In South Africa, long-proboscid fly species are active only for short periods of the year, thus creating strong temporal niche gradients [26].

## 5. Intraspecific trait–environment correlations

Trait–environment correlations provide key evidence for niche-driven diversification, and hence ecotypes, in plants and animals [8], although they need to be interpreted cautiously. These correlations are most compelling when multiple species distributed along the same environmental gradient exhibit similar patterns of intraspecific divergence and convergence in floral traits [51,53].

Flower depth (or floral tube length) is the most thoroughly studied floral trait in terms of local adaptation to pollinators. Darwin [80] proposed that in environments with long-proboscid flower visitors, selection through mechanical fit that promotes pollen transfer will favour floral tubes that exceed proboscis length. In contrast, if predominant flower visitors have short proboscides, selection may favour shorter floral tubes that allow these visitors to reach nectar and forage consistently. Geographical correlations between functional flower depth and proboscis length occur widely, including in species pollinated by bees [81], hawkmoths [62,76,82] (figure 1a–d), butterflies [23], long-proboscid flies [51,53] and birds [60,83], as well as those pollinated by different pollinator functional groups in different regions [16,59,60,84] (figure 1e–f).

Flower size is expected to be correlated with the body size of pollinators, particularly when it influences mechanical fit [85]. Flower size in *Calceolaria polyrhiza* (Calceolariaceae) varies among populations in Patagonia with the size of oil-collecting bees and is decoupled from plant traits that correlate with climate factors [86]. Flower size and bee body size have also been found to co-vary along elevational gradients in Europe [87,88]. However, bees are usually sampled on flowers in these studies and therefore whether bee body size is filtered by floral traits, reflects competition among plants or represents an independent niche variable is generally not known.

Flower colour also often varies geographically in association with different local pollination conditions. In some food-deceptive orchids, flower colour varies among populations in association with the dominant colour in local assemblages of rewarding food plants as pollinators learn to prefer those colours (figure 1g). More commonly, flower colour varies according to different pollinator assemblages. For example, in western North America *Mimulus aurantiacus* (Phrymaceae), has an eastern ecotype (figure 1h) with yellow flowers, inserted stamens and is pollinated mainly by hawkmoths, whereas a western ecotype (figure 1i) has red flowers with exserted stamens and is pollinated mostly by hummingbirds [63]. Similarly, in the Cape region of South Africa bird-pollinated ecotypes of *Erica plukenetii* (Ericaceae) have red flowers, whereas the moth-pollinated ecotype has white flowers [60] (figure 1j) and geographically structured flower colour variation in *Drosera cistiflora* (Droseraceae) is associated with switches among different hopliine beetle assemblages [89]. In *Papaver rhoeas* (Papaveraceae), populations in the Mediterranean basin have red UV-absorbing flowers and are pollinated by glaphyrid beetles, whereas populations in central Europe, where these beetles do not occur, have red UV-reflecting flowers that are pollinated by bees [90].

Scent is an important floral signal in many pollination systems and varies among populations in many plant species [91]. In some cases, scent varies with pollinator assemblage [62,77,92,93]. For species that attract pollinators almost entirely with scent, such as those with sexually deceptive flowers that attract male Hymenoptera, scent chemistry can diverge through adaptation to different pollinators without marked changes in floral morphology. The evolution of such cryptic pollination ecotypes has been reported for several species in the Australian sexually deceptive orchid genus *Drakaea* [94–96].

Floral traits often vary among populations in association with the incidence of autonomous self-fertilization [97–100]. Plants with a capacity for autogamy often exhibit a ‘selfing syndrome’ that includes smaller flowers with limited herkogamy and reduced pollen production [36]. Geographical variation in the ability of plants to self-pollinate often correlates with pollinator availability [97,100–102]. In distylous and tristylous plant species that typically exhibit strong herkogamy, colonization of habitats with pollinator deficits, such as islands, higher elevations and range margins, is often accompanied by the evolution of self-pollinating ‘homostyles’ with reduced herkogamy [37,98]. In these lineages, the heteromorphic incompatibility system was usually lost prior to the evolution of reduced herkogamy. For example, *Primula oreodoxa* in China is self-compatible and lowland populations maintain reciprocal herkogamy with high rates of pollinator visitation, whereas homostyly and smaller flowers (figure 1k) have evolved at higher elevations (1600−2500 m) where effective long-tongued pollinators are scarce [37]. Selfing rates in homostylous populations were twice that in distylous populations [37]. *Primula dumicola* (figure 1k), another member of the species complex that occurs at even higher elevations, is highly selfing and even smaller flowered, suggesting that the evolution of selfing ecotypes in this lineage promoted speciation. This is consistent with numerous shifts from distyly to homostyly and speciation in this section of *Primula* [103].

Plants that experience pollinator deficits in parts of their geographical range may also show partial or complete shifts to wind pollination [104], a transition that has occurred frequently in the angiosperm radiation. These transitions probably occur via the intermediate stage of ambophily (mixed animal and wind pollination) and in *Thalictrum* (Ranunculaceae), this shift appears to be characterized by clinal variation among populations, rather than discrete ecotypes [105].

## 6. Experimental demonstration of pollination ecotypes

Growing plants from different populations in a common garden [1] or a single genotype in a range of environments [2] are the simplest ways to establish the relative genetic and environmental contributions (through plasticity) to variation in floral traits [106]. In general, floral traits seem to be more canalized than vegetative traits [107] and floral trait differences among populations are often maintained in a common garden [63,89,108], even for physiological traits such as scent chemistry [109]. Although floral traits are generally heritable [34,85,110], they can exhibit some phenotypic plasticity, which in itself could be locally adaptive [106,111,112]. Common garden experiments are usually not feasible for plants that are difficult to cultivate, such as most terrestrial orchids, but the genetic basis of phenotypic differences among populations can sometimes be inferred when ecotypes grow together in contact zones under similar environmental conditions [62,77].

Local adaptation of both plant and animal species is commonly assessed by reciprocal translocation of individuals from contrasting environments to test the prediction that local genotypes outperform foreign genotypes [113]. To date, only a few studies have used this approach to test for local adaptation in floral traits. Consistent with local adaptation, some of these tests found that local forms outperformed foreign forms with respect to pollen deposition and seed production [114] or rate of pollinator visitation [78,96]. In other cases, one form outperformed the other in both environments or non-reciprocally in just one environment [76,100,108,115]. These results suggest either maladaptiveness of one form or that an experiment conducted in a single season may not reflect the cumulative effect of natural selection in variable pollination environments.

Although reciprocal translocation experiments provide valuable information concerning local adaptation, they can be challenging to conduct and interpret, particularly when used to evaluate pollination ecotypes. Ethically, they are not advisable for rare species or when contamination of local gene pools is possible [113]. Emasculation of flowers can ameliorate the latter problem, but it can skew outcomes if it affects pollinator behaviour, especially in systems serviced by pollen-collecting insects. Translocated plants also often flower poorly [23] and experimental plants introduced to a site are often outnumbered by local plants in the surrounding community. Consequently, introduced plants may attract fewer pollinators because pollinators are conditioned on the local plants, rather than because traits of the introduced plants are mismatched with local pollinators. To address this potential problem, Waterman *et al*. [65] conducted an additional array of experiments at sites where bees lacked experience with either floral form and found home site fitness advantages for local forms that were similar to those at sites where bees had experience with local forms. Introduced plants may also produce fewer seeds if they receive pollen from a different part of the pollinator’s bodies than do local plants, or if they are not fully interfertile with local plants. Finally, the seed set may poorly represent pollination success if it is limited by resources. This possibility can be assessed by supplemental hand pollination to show that levels of seed set reflect pollination processes and not simply resource availability [100,116].

## 7. Divergent pollinator-mediated selection on floral phenotypes

In addition to demonstrating local adaptation in translocation studies, the phenotypic targets of selection need to be identified [34]. Few studies have tested whether divergent selection acting on natural phenotypic variation can explain observed variation among populations. Most of these studies have tested for selection on floral tube depth [51,62,114] and at least one study detected divergent selection on floral scent traits [93]. Some studies have also tested for divergent selection on traits involved in self-fertilization. For example, Moeller & Geber [42] found stronger phenotypic selection for reduced herkogamy, protandry and reduced petal size in *Clarkia xantiana* in small populations lacking congeners that facilitate pollinator visitation than in populations with congeners. Identifying the phenotypic targets of selection involves several challenges. Paradoxically, when selection has been strong historically, phenotypic variation in populations is often small and selection harder to detect [34]. In addition, the effects of correlated traits can be difficult to untangle. Although phenotypic selection studies commonly include several traits and use partial regression coefficients to isolate selection on particular traits, selection on correlated unmeasured traits could account indirectly for observed selection.

To overcome these problems, many authors have experimentally manipulated traits to recreate the likely range of phenotypic variation present in ancestral populations or that could arise through gene flow from neighbouring populations [77,117–119]. The functional significance of floral traits can also be tested using artificial flowers that allow comparison of the effects of contrasting traits such as colour or spur length while controlling for other potential cues [78,118]. Testing the responses of pollinators to scent volatiles in olfactometers or other bioassay experiments can also reveal which compounds mediate pollinator shifts [77,92]. Additionally, arrays of plants from different geographical regions can be used to test how selection might act when greater phenotypic variation is available or if new phenotypes were introduced by immigration [63,65,120]. Using arrays of F_2_ plants from hybrid crosses is another good method for introducing a realistic range of phenotypic variation to test the potential for divergent selection [100,121]. Finally, manipulation of the entire pollinator assemblage in controlled environments [110] can be used to assess how selection could account for the observed geographical pattern in phenotypes [85].

## 8. From ecotypes to species

Two very different perspectives prevail among biologists concerning the relation between micro- and macroevolution. In Darwin’s view, microevolution, such as the formation of new morphotypes (his ‘varieties’), involves the same process that gives rise to species differences. Consequently, he considered that varieties and species differed only in the extent of phenotypic discontinuities, which he attributed mainly to the extinction of intermediates [122]. Clausen and other biosystematists expressed similar views, but were also informed by genetic concepts developed during the evolutionary synthesis, including the importance of reproductive isolation (and thus also allopatry) for genetic divergence and the possibility of phenotypic changes due to neutral processes [2,3,5,18,22]. Clausen and others argued that post-mating genetic incompatibilities arose slowly and were often not present during the divergence of ‘ecospecies’ that exhibit mainly ecogeographical isolation [22]. This view of (non-polyploid) speciation as gradual and occurring locally via innumerable branching (and extinctions) of new varieties is also congruent with evidence for limited ‘swamping’ gene flow among populations of many plants and animals [123].

The other perspective, championed by Mayr [124], was that microevolution usually occurs after speciation for reasons that may be unconnected with lineage splitting (considered synonymous with speciation). Phenotypic divergence was thus mainly viewed as due to speciation, rather than the converse. Mayr’s premise was that gene flow among populations was extensive enough for species to evolve collectively through anagenesis unconnected with speciation. Adopting this perspective, Coyne & Orr [125, p. 11] stated that ‘Darwin conflated the problem of change within a lineage with the problem of the origin of new lineages’.

**Figure 2.**
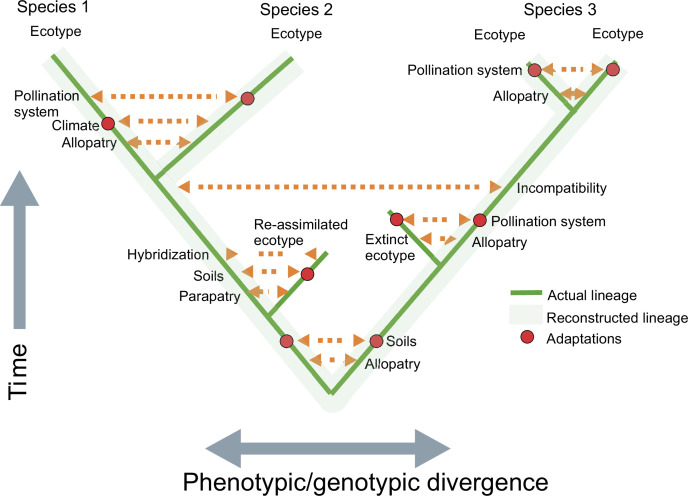
A schematic representation of the process of speciation through progressive intermediate stages of ecotype formation. Initial allopatry or parapatry of population clusters allows local adaptation to various environmental conditions. Resulting ecotypes can merge back into parental lineages, become extinct or persist to diverge further, ultimately becoming species. The sequences of adaptations shown are hypothetical and in some cases are confined to one lineage, as expected in peripatric divergence. Note that species 1 and 2 could also be interpreted as ecotypes of a single species. Adapted from van der Niet *et al.* [25].

These divergent perspectives were largely reconciled with the contemporary development of models of ecological speciation [8]. The ‘Grant–Stebbins model’ of pollinator-driven speciation [26] is an example of this process (figure 2). It places equal emphasis on adaptive niche-driven processes that lead to phenotypic diversification and the isolating barriers that accumulate during niche specialization. Ecotype formation can theoretically even occur in parapatry in the face of gene flow if selection differentials exceed rates of migration into populations [14,17,63].

In the *Origin*, Darwin [125, p. 56] noted that ‘varieties or incipient species’ occur most often in large genera and he attributed this to ongoing diversification. To test this idea, one could more specifically ask whether the pollinator shifts and floral diversification that characterize certain genera are also manifest at the intraspecific level. This approach has yet to be applied formally, but, strikingly, intraspecific pollinator shifts seem particularly common in lineages with frequent shifts at the macroevolutionary level. Families and genera for which this has been documented include: Iridaceae*—Lapeirousia* [51] and *Gladiolus* [62]; Orchidaceae*—Disa* [117], *Platanthera* [76], *Ophrys* [120] and *Drakaea* [95]; Phrymaceae*—Mimulus* (Phrymaceae) [63]; Primulaceae*—Primula* [56,58]; and Ericaceae*—Erica* [60]. Correlations between micro- and macroevolutionary trait divergence have been reported more generally for a wide range of lineages [126]. These findings are consistent with the expectation that ‘macroevolutionary patterns represent the aggregate outcomes of microevolutionary processes at the intraspecific level’ [61, p. 279].

## 9. Pollination ecotypes and reproductive isolation

Clausen considered ecotypes to be stages in the formation of species and maintained that the ‘evolutionary barriers to the interchange of heredities evolve gradually’ [22, p. 86]. He also argued that younger sister species (‘ecospecies’) tend to have only partial genetic incompatibilities and often hybridize at contact zones, whereas older and more distantly related species have stronger genetic incompatibilities and can occur without admixture in the same habitats [22] (figure 2). Clausen’s view that ‘evolution is reticulate until the level of the genus’ [22, p. 86] is remarkably prescient, given the recent findings from genetic studies [127]. Similarly, Mallet [12, p. 502] argued that ‘reproductive isolation thus is not suddenly complete at the species boundary, but merely declines exponentially with genetic divergence across it’.

Despite pollination ecotypes tending to occupy separate geographical ranges, often in strict allopatry [23,25,51,78,86,92,100,117], there is a common view that ecotypes arise in parapatry with gene flow [14]. This idea probably arose because studies of ecotypes often focus on cases with contact zones [128]. Some studies have shown that differences in pollination systems can contribute strongly to reproductive isolation among ecotypes or recently diverged species pairs [120,128,129], but in most cases, this applies only in a narrow contact zone and the main isolating barrier is ecogeographical [128].

## 10. Species concepts

Darwin did not consider species to be special units of organization in nature. Instead, he considered discontinuities among species to be mainly due to extinctions, arguing that ‘the only distinction between species and well-marked varieties is, that the latter are known, or believed, to be connected at the present day by intermediate gradations, whereas species were formerly thus connected’ [130, p. 485].

Early work on ecotypes during the Clausen era drew on Darwin’s emphasis on the importance of varieties arising from natural selection and Dobzhansky’s [21] arguments about the importance of reproductive isolating barriers. However, the biological species concept (BSC), as formulated by Mayr, became increasingly focused on isolating barriers as the *sine qua non* of species identity. With genetic evidence now mounting for the gradual evolution of reproductive isolating barriers, both below and above the species level [11,12], the case for species as a special category in nature has weakened. Indeed, the pendulum appears to have swung again in favour of the value of investigating ecotypes (and intraspecific forms more generally) as part of the continuum of divergence. One consequence of this continuum is the difficulty of deciding whether or not allopatric ecotypes and even those that hybridize in narrow contact zones constitute ecotypes or distinct species [11,12,25,63,77]. Some researchers have advocated the use of DNA barcoding and multi-locus coalescent techniques to identify species [131]. However, this can divorce the origin of species from any role for natural selection and even runs counter to the BSC, since fully inter-fertile forms that are separated only by geography are not immune from being raised to the species level if they have sufficient genetic structure [131,132].

Reproductive isolating barriers, whether simply due to geography (i.e. fully extrinsic) or based on ecology or genetic incompatibilities, are obviously critical for the accumulation of lineages and species coexistence, but they are not always useful for delimiting species. These barriers also exist among populations within species. ‘Ecogeographic’ isolation, for example, probably occurs commonly among clusters of populations in wide-ranging plant species that occur in different soils and climates.

Using a niche-based concept to define species [133] is also unsatisfactory because every ecotype or population along an ecocline would be a species. Van Valen [133, p. 236] was aware of this difficulty and considered ecotypes a potential ‘mechanism of origin for species’. Niche shifts certainly characterize many adaptive radiations [8], but they clearly arise below the species level and do not constitute the species level *per se*.

Depending on their research interests, biologists have variously emphasized the importance of many different properties as features distinguishing species, including phenotypic and genetic discontinuities, niche divergence, reproductive isolating barriers, and monophyly. However, no single property can be used to define species in all cases [134]. For example, researchers studying hybrid zones tend to emphasize the role of isolating barriers in the context of the BSC, but this has limited power to explain the ‘origins’ of species. In contrast, the study of ecotypes across the entire range of species identifies the environmental drivers of diversification in addition to the implications of the adaptive divergence for reproductive isolation. Encouragingly, some recent definitions of speciation recognize the multifaceted properties of species. Maddison & Whitton [135, p. 23], for example, defined the process of speciation as ‘the set of processes by which phylogenetic communities derived from a recent common ancestor diverge and gain distinction in genes and traits (including, in sexuals, those concerning interbreeding), from the initial stages of separation and divergence, to the widespread establishment of distinctive traits, interactions and ecologies’. This description is consistent with the role of ecotypes in the speciation process and in species concepts.

## 11. Why natural history matters

Insufficient natural history data have consistently been identified as a major rate-limiting factor for understanding the diversification of plants [38,136] and animals [137]. Despite the availability of complete phylogenies and trait information for the extant species in many large genera, the environmental drivers of diversification remain poorly understood because of insufficient information about the interactions of species with their environments. Obtaining these data, particularly for tropical regions, is extremely challenging. Work on natural history is often perceived as ‘old-fashioned’ and does not easily attract funding, yet it remains essential for the interpretation of trait evolution.

The study of pollination ecotypes is best undertaken through investigations conducted in multiple populations throughout the geographical range of the species. Unfortunately, much of the current natural history work on pollination systems is limited to the study of networks of interactions among plants and flower visitors at single locations. What is also often lacking are detailed studies of floral traits, together with estimates of pollinator importance (the product of their visitation rate and effectiveness as pollen vectors [138]), and experiments to test whether plants rely on pollinators for seed production. Detailed case studies are needed as a source of data for comparative biology at both the ecotype and species levels and remain essential for gaining insights into the reproductive biology and diversification of plant species.

## 12. Conclusions

The origin of most plant species results from local adaptation to environmental niches. Over time, this process can lead to the development of ecotypes, some of which diversify further until the resulting forms are sufficiently distinct to be recognized as species (figure 2). The accumulation of various reproductive isolating barriers allows some of these distinct forms to persist in new lineages and ultimately become new species. However, most ecotypes probably either become extinct and represent dead ends in the tree of life or are re-assimilated into other ecotypes (figure 2). Extinction of intermediate forms is the most likely (and overlooked) explanation for the marked discontinuities among extant species, including many ‘sister species’. By studying pollination ecotypes, researchers can gain valuable insights into the processes and mechanisms that underlie floral diversification, the hallmark of angiosperm radiation.

## Data Availability

This article has no additional data.
